# Frequency and predisposing factors for canine otitis externa in the UK – a primary veterinary care epidemiological view

**DOI:** 10.1186/s40575-021-00106-1

**Published:** 2021-09-07

**Authors:** Dan G. O’Neill, Andrea V. Volk, Teresa Soares, David B. Church, Dave C. Brodbelt, Camilla Pegram

**Affiliations:** 1 Pathobiology and Population Sciences, The Royal Veterinary College, Hawkshead Lane, North Mymms, Hatfield, Herts AL9 7TA UK; 2 Department of Small Animal Medicine and Surgery, University of Veterinary Medicine Hannover, Foundation, Buenteweg 9, 30559 Hannover, Germany; 3 Clinical Science and Services, The Royal Veterinary College, Hawkshead Lane, North Mymms, Hatfield, Herts AL9 7TA UK

**Keywords:** VetCompass, Electronic patient record, Breed, Dog, Epidemiology, Primary-care, Purebred, Pendulous ear, Erect ear

## Abstract

**Background:**

Otitis externa is a commonly diagnosed disorder in dogs and can carry a high welfare impact on affected animals. This study aimed to report the prevalence and explore the role of breed and aural conformation as predisposing factors for canine otitis externa in the UK. The study used a cohort design of dogs under UK primary veterinary care at clinics participating in the VetCompass Programme during 2016. Risk factor analysis used multivariable logistic regression modelling.

**Results:**

The study included a random sample of 22,333 dogs from an overall population of 905,554 dogs under veterinary care in 2016. The one-year period prevalence of otitis externa was 7.30% (95% confidence interval [CI]: 6.97 to 7.65). Breed and ear carriage were the highest ranked risk factors. Compared with crossbred dogs, sixteen breed types showed increased odds, including: Basset Hound (odds ratio [OR] 5.87), Chinese Shar Pei (OR 3.44), Labradoodle (OR 2.95), Beagle (OR 2.54) and Golden Retriever (OR 2.23). Four breeds showed protection (i.e. reduced odds) of otitis externa: Chihuahua (OR 0.20), Border Collie (OR 0.34), Yorkshire Terrier (OR 0.49) and Jack Russell Terrier (OR 0.52). Designer breed types overall had 1.63 times the odds (95% CI 1.31 to 2.03) compared with crossbred dogs. Compared with breeds with erect ear carriage, breeds with pendulous ear carriage had 1.76 times the odds (95% CI 1.48 to 2.10) and breeds with V-shaped drop ear carriage had 1.84 times the odds (95% CI 1.53 to 2.21) of otitis externa.

**Conclusions:**

Breed itself and breed-associated ear carriage conformation are important predisposing factors for canine otitis externa. Greater awareness of these associations for both predisposed and protected breeds could support veterinary practitioners to promote cautious and low-harm approaches in their clinical advice on preventive care for otitis externa, especially in predisposed breeds.

## Plain English Summary

Otitis externa is a common and often painful condition of the ears of dogs. The probability of otitis externa varies widely between dogs depending on their tendency towards other skin problems such as allergic skin disease. However, breed-related anatomical features such as the degree and point of folding of the ear flap or the overall size of the dog can act to further predispose towards otitis externa. Greater understanding of these predisposing factors could lead to opportunities for breeders to breed away from predisposing features and also help owners and veterinarians to predict which dogs are most at risk of this painful condition.

Veterinarians record clinical notes when dogs visit for veterinary care, and these clinical notes can act as a rich source of information on the health of these dogs. This study accessed anonymised clinical records of almost a million dogs in the UK to identify dogs diagnosed with otitis externa. The breed, age, bodyweight and other information available on these dogs were used to explore why certain dog types were predisposed to otitis externa.

A random sample of 22,333 dogs was selected for analysis. Of these, 7.30% (over 7 in a hundred) were diagnosed with otitis externa in a single year. There were 16 breeds with higher risk of otitis externa compared with crossbred dogs. Breeds with the highest risk were Basset Hound (× 5.87), Chinese Shar Pei (× 3.44), Labradoodle (× 2.95), Beagle (× 2.54) and Golden Retriever (× 2.23). Four breeds showed reduced risk: Chihuahua (× 0.20), Border Collie (× 0.34), Yorkshire Terrier (× 0.49) and Jack Russell Terrier (× 0.52). Breeds with pendulous ear carriage (× 1.76) and V-shaped drop ear carriage (× 1.84) had higher risk of otitis externa compared with breeds with erect ear carriage.

Breed itself and also breed-associated ear carriage conformation are associated with differing probability of otitis externa in dogs. Better understanding about how these factors affect the probability of otitis externa in dogs can help veterinary practitioners to promote cautious and low-harm approaches to preventing otitis externa (e.g., by advocating ear cleaning with a dry paper cloth, judicious use of ear cleaners with antimicrobial properties and avoidance of overzealous ear-cleaning) and can also assist breeders to breed away from features of dogs that predispose to otitis externa.

## Background

Otitis externa describes an inflammatory state of the outer ear canal, with or without pinnal involvement [1]. The external ear canal is lined with epithelial cells, comparable to the ubiquitous epidermis, with variable hair follicles [2, 3], sebaceous and ceruminous glands [4], and is populated with a microflora [5]. All these anatomical generalities vary widely between breeds, suggesting that breed should be considered as an important predisposing factor for otitis externa in dogs [2, 6]. Otitis externa cases present across a wide clinical spectrum, ranging from acute inflammatory or inflammatory/infectious to chronic cases, with or without middle ear involvement, and may also be associated with hyperplastic or neoplastic changes [7, 8]. The aetiology of otitis externa cases is generally multifactorial and has been classified according to the PSPP-system: primary, secondary, predisposing and perpetuating factors. Primary factors, such as allergic skin disease, endocrinopathies, and keratinisation and immune-mediated disorders, initiate inflammation/infection of the skin and thus the ear canal, while other primary factors such as foreign bodies can affect just the ear canal itself. In contrast, predisposing factors on their own, such as swimming, humidity and pinna conformation, contribute to rather than initiate the otitis externa problem. Within the pathogenesis of otitis externa, secondary (e.g., infectious) factors and perpetuating factors (e.g., chronic changes within ear canal, tympanic membrane and middle ear) contribute to the ongoing pathogeneis of otitis externa [7, 9]. Specific focus on predisposing factors to identify both predispositions (i.e., higher odds) and protections (i.e. reduced odds) would be of major interest for breeders and animal welfare scientists and could lead to applications in breeding programs to lower the risk for dogs acquiring otitis externa [10, 11].

The reported prevalence of otitis externa in dogs ranges from 8.7% [12] to 20% [8, 13] in Europe, depending on the study design. Otitis externa was reported in 10.2% of dogs under primary veterinary care in the UK, and was the most prevalent disorder overall [14]. Otitis externa has been reported with consistently high prevalence in several breed-specific descriptive studies [15–19]. However, the univariable analytical methods applied in these studies did not allow for deeper comparison of risk between breeds after accounting for possible confounding factors such as age, sex, neuter status and insurance [20, 21]. There are few reports that explore association between age and otitis externa [22], although a study on 149 dogs did demonstrate differences in distribution of pathogens at different ages of onset of otitis externa [23]. Likewise, there are limited reports exploring association between sex and otitis externa, although a report based on 273 dogs presenting to teaching and referral hospitals in India identified higher prevalence of otitis externa in male dogs compared to females [24].

Previously proposed predisposing factors include conformation of the pinna and ear canal, as well as the numbers of hair follicles within these structures [7]. There is good evidence that exposure to moisture, for example in dogs that regularly swim or hunt, or breeds with anagen hair coats that are frequently bathed and groomed, acts as a predisposing factor to water-induced, humidity- or foreign body-related ear problems [7, 25]. Other proposed predisposing factors include excessive cerumen production (often breed related), obstructions to the physiological air flow within the external ear canal, alterations of the normal microflora within the canal (e.g., due to disease, preceding therapies), irritant iatrogenic/owner-related applications (e.g., cleaners, cotton tips) and systemic debilitation (Griffin 2010). The current study aimed to apply a quantitative methods approach based on the large volume of data available within VetCompass to explore predisposing factors for canine otitis externa in the UK. Breed-related aspects as described above can act as predisposing factors promoting otitis externa, even in the absence of primary systemic skin diseases [7, 26]. Providing evidence on breed and conformational predisposing factors could support the work of breeders who prioritise health in their dogs to breed towards dog types with reduced risk of otitis externa. Novel information on predisposing factors may be especially relevant at the current time with the advent of rising popularity of a range of designer breeds that have largely unknown health status [27].

The clinical management of chronic otitis externa is often a highly frustrating, time-consuming and expensive endeavour between veterinary surgeon, owner and patient [28]. Improved understanding of predisposing factors in high-risk dogs should lead to better detection of cases of otitis externa and improved owner compliance with preventive and therapeutic care. Otitis externa is recognised as a disorder that carries a high negative welfare impact for affected dogs. A primary veterinary care study that scored the severity of eight common disorders in dogs ranked otitis externa as the second most severe of these disorders [29]. Quality of life for affected dogs is reduced by their pruritus and pain [29], but may be further affected by uncomfortable otorrhoea, malodour, partial to complete hearing loss, and severe pain due to ulcerations or deeper and more marked inflammation of middle ears and surrounding structures (e.g., temporomandibular joint) [28, 30–32]. Some affected dogs may require radical surgical interventions [33]. Loss of hearing and long-term low- to high-grade otic pain from chronic otitis externa are often overlooked as an animal welfare issue because both are unfortunately not so obvious to owners or veterinarians as are acute purulent discharge, malodour or redness of the ear [30, 31].

Using anonymised primary care veterinary clinical data from the VetCompass™ Programme [34], the current study aimed to report the prevalence of diagnosis of otitis externa in dogs overall and within commonly affected breeds. The study also aimed to investigate population-based statistical associations to explore signalment and conformation as predisposing factors for otitis externa. It is acknowledged that associations reported here for predisposing factors may be complicated to differing degrees by additional primary, secondary and perpetuating factors for otitis externa in some individual dogs [8, 25]. This study did not aim to report on comorbidity of otitis externa with other aural or skin disorders, or on the clinical management or outcomes for otitis externa.

## Results

### Prevalence

**Fig. 1:**
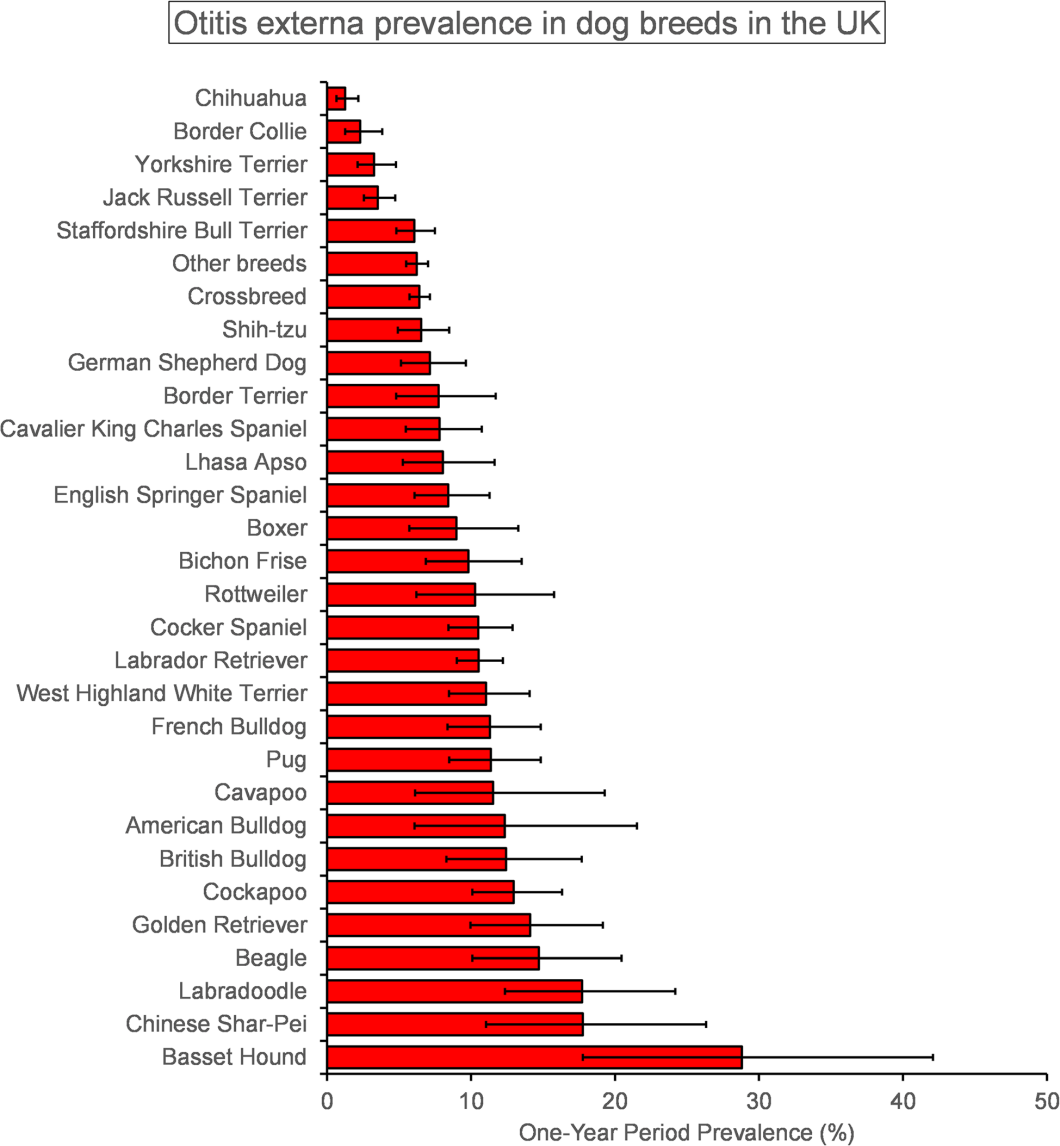
One-year (2016) period prevalence for otitis externa in dog breeds under primary veterinary care in the VetCompass™ Programme in the UK

The study included a random sample of 22,333 dogs (2.47%) from an overall population of 905,554 dogs under veterinary care in 2016 attending 784 veterinary clinics participating in VetCompass. There were 1631/22,333 otitis externa cases identified during 2016, yielding a one-year period prevalence of 7.30% (95% CI: 6.97–7.65). The breed types with the highest otitis externa prevalence were Basset Hound (28.81%, 17.76–42.08), Chinese Shar Pei (17.76%, 11.04–26.33), Labradoodle (17.71%, 12.36–24.19), Beagle (14.72%, 10.09–20.45), Golden Retriever (14.11%, 9.97–19.15) and Cockapoo (12.97%, 10.09–16.32). The breeds with the lowest otitis externa prevalence were Jack Russell Terrier (3.53%, 2.56–4.74), Yorkshire Terrier (3.27%, 2.13–4.79), Border Collie (2.30%, 1.26–3.83) and Chihuahua (1.26%, 0.65–2.18) (Fig. 1).

Of the otitis externa cases with data available for that variable, 1192 (73.26%) were purebred, 689 (42.32%) were female and 820 (50.37%) were neutered. Dogs with otitis externa had a median adult bodyweight of 17.00 kg (IQR: 10.16–28.70, range 2.26–78.00) and median age was 4.72 years (IQR: 2.18–8.18, range 0.23–17.70). The most common breed types among the otitis externa cases were Labrador Retriever (154, 9.44%), Cocker Spaniel (81, 4.97%), Staffordshire Bull Terrier (79, 4.84%) and Cockapoo (62, 3.80%), along with crossbred dogs (302, 18.52%) (Tables 1 and 2).

**Table 1:** Descriptive and univariable logistic regression results for breed-related factors as predisposing factors for otitis externa during 2016 in dogs under primary veterinary care in the VetCompass™ Programme in the UK. Column percentages shown in brackets

Variable	Category	Case No. (%)	Non-case No. (%)	Odds ratio	95% CI^a^	Category *P*-value	Variable *P*-value
Purebred status	Crossbred	302 (18.56)	4408 (21.36)	Base			< 0.001
	Designer	133 (8.17)	1162 (5.63)	1.67	1.35–2.07	< 0.001	
	Purebred	1192 (73.26)	15,064 (73.01)	1.15	1.01–1.32	0.031	
Breed	Crossbreed	302 (18.52)	4408 (21.29)	Base			< 0.001
	Basset Hound	17 (1.04)	42 (0.20)	5.91	3.32–10.50	< 0.001	
	Chinese Shar Pei	19 (1.16)	88 (0.43)	3.15	1.89–5.24	< 0.001	
	Labradoodle	31 (1.90)	144 (0.70)	3.14	2.10–4.71	< 0.001	
	Beagle	29 (1.78)	168 (0.81)	2.52	1.67–3.80	< 0.001	
	Golden Retriever	34 (2.08)	207 (1.00)	2.40	1.64–3.51	< 0.001	
	Cockapoo	62 (3.80)	416 (2.01)	2.18	1.63–2.91	< 0.001	
	English Bulldog	26 (1.59)	183 (0.88)	2.07	1.35–3.18	0.001	
	American Bulldog	10 (0.61)	71 (0.34)	2.06	1.05–4.03	0.036	
	Cavapoo	12 (0.74)	92 (0.44)	1.90	1.03–3.51	0.039	
	Pug	47 (2.88)	366 (1.77)	1.87	1.35–2.59	< 0.001	
	French Bulldog	45 (2.76)	353 (1.71)	1.86	1.34–2.59	< 0.001	
	West Highland White Terrier	57 (3.49)	459 (2.22)	1.81	1.34–2.44	< 0.001	
	Labrador Retriever	154 (9.44)	1308 (6.32)	1.72	1.40–2.11	< 0.001	
	Cocker Spaniel	81 (4.97)	690 (3.33)	1.71	1.32–2.22	< 0.001	
	Rottweiler	18 (1.10)	157 (0.76)	1.67	1.01–2.76	0.044	
	Bichon Frise	33 (2.02)	303 (1.46)	1.59	1.09–2.32	0.016	
	Boxer	22 (1.35)	223 (1.08)	1.44	0.92–2.27	0.115	
	English Springer Spaniel	40 (2.45)	435 (2.10)	1.34	0.95–1.89	0.094	
	Lhasa Apso	25 (1.53)	286 (1.38)	1.28	0.83–1.95	0.261	
	Cavalier King Charles Spaniel	34 (2.08)	401 (1.94)	1.24	0.86–1.79	0.258	
	Border Terrier	20 (1.23)	238 (1.15)	1.23	0.77–1.96	0.395	
	German Shepherd Dog	39 (2.39)	507 (2.45)	1.12	0.79–1.59	0.512	
	Shih Tzu	52 (3.19)	743 (3.59)	1.02	0.75–1.38	0.891	
	Other purebreds	250 (15.33)	3764 (18.18)	0.97	0.82–1.15	0.725	
	Staffordshire Bull Terrier	79 (4.84)	1225 (5.92)	0.94	0.73–1.22	0.643	
	Jack Russell Terrier	42 (2.58)	1148 (5.55)	0.53	0.38–0.74	< 0.001	
	Yorkshire Terrier	25 (1.53)	740 (3.57)	0.49	0.33–0.75	0.001	
	Border Collie	14 (0.86)	594 (2.87)	0.34	0.20–0.59	< 0.001	
	Chihuahua	12 (0.74)	943 (4.56)	0.19	0.10–0.33	< 0.001	
Kennel Club Recognised Breed	Not recognised	463 (28.46)	5976 (28.96)	Base			0.665
	Recognised	1164 (71.54)	14,658 (71.04)	1.02	0.92–1.15	0.666	
Kennel Club Breed Group	Breed not Kennel Club recognised	463 (28.46)	5976 (28.96)	Base			< 0.001
	Toy	165 (10.14)	3148 (15.26)	0.68	0.56–0.81	< 0.001	
	Utility	222 (13.64)	2296 (11.13)	1.25	1.06–1.47	0.009	
	Terrier	236 (14.51)	3412 (16.54)	0.89	0.76–1.05	0.171	
	Gundog	340 (20.90)	2945 (14.27)	1.49	1.29–1.73	< 0.001	
	Hound	71 (4.36)	727 (3.52)	1.26	0.97–1.64	0.083	
	Pastoral	66 (4.06)	1259 (6.10)	0.68	0.52–0.88	0.004	
	Working	64 (3.93)	871 (4.22)	0.95	0.72–1.24	0.701	
Ear carriage	Erect	220 (13.49)	3898 (18.83)	Base			< 0.001
	Semi-erect	276 (16.92)	4654 (22.48)	1.05	0.88–1.26	0.594	
	V-shaped drop	299 (18.33)	2734 (13.21)	1.94	1.62–2.32	< 0.001	
	Pendulous	395 (24.22)	3752 (18.12)	1.87	1.57–2.21	< 0.001	
	Variation	441 (27.04)	5664 (27.36)	1.38	1.17–1.63	< 0.001	
Skull conformation	Dolichocephalic	134 (8.24)	1610 (7.81)	Base			0.941
	Mesocephalic	754 (46.37)	9587 (46.49)	0.94	0.78–1.14	0.562	
	Brachycephalic	303 (18.63)	3866 (18.75)	0.94	0.76–1.16	0.578	
	Not categorised	435 (26.75)	5559 (26.96)	0.94	0.77–1.15	0.548	
Poodle	Not poodle-type	1188 (72.84)	15,302 (73.92)	Base			< 0.001
	Poodle-type	137 (8.40)	924 (4.46)	1.91	1.58–2.31	< 0.001	
	Not categorised	306 (18.76)	4476 (21.62)	0.88	0.77–1.00	0.055	
Spaniel	Non spaniel-type	1152 (70.63)	14,539 (70.23)	Base			< 0.001
	Spaniel-type	173 (10.61)	1687 (8.15)	1.29	1.09–1.53	0.003	
	Not categorised	306 (18.76)	4476 (21.62)	0.86	0.76–0.98	0.027	

^a^CI confidence interval

**Table 2:** Descriptive and univariable logistic regression results for non-breed-related signalment factors as predisposing factors for otitis externa during 2016 in dogs under primary veterinary care in the VetCompass™ Programme in the UK. Column percentages shown in brackets

Variable	Category	Case No. (%)	Non-case No. (%)	Odds ratio	95% CI^a^	Category *P*-value	Variable *P*-value
Adult (> 18 months) bodyweight (kg)	< 10.0	299 (18.33)	5115 (24.71)	Base			< 0.001
	10.0 - < 15.0	261 (16.00)	2203 (10.64)	2.03	1.70–2.41	< 0.001	
	15.0 - < 20.0	144 (8.83)	1550 (7.49)	1.59	1.29–1.95	< 0.001	
	20.0 - < 25.0	143 (8.77)	1461 (7.06)	1.67	1.36–2.06	< 0.001	
	25.0 - < 30.0	116 (7.11)	1234 (5.96)	1.61	1.29–2.01	< 0.001	
	30.0 - < 40.0	194 (11.89)	1575 (7.61)	2.11	1.74–2.55	< 0.001	
	≥ 40.0	80 (4.90)	549 (2.65)	2.49	1.92–3.24	< 0.001	
	Unavailable	394 (24.16)	7015 (33.89)	0.96	0.82–1.12	0.612	
Bodyweight relative to breed mean	Lower	560 (34.33)	7486 (36.16)	Base			< 0.001
	Equal/Higher	673 (41.26)	6155 (29.73)	1.46	1.30–1.64	< 0.001	
	Unavailable	398 (24.40)	7061 (34.11)	0.75	0.66–0.86	< 0.001	
Age (years)	< 1.0 years	116 (7.11)	2392 (11.55)	Base			< 0.001
	1.0 - < 2.0 years	251 (15.39)	3027 (14.62)	1.71	1.36–2.14	< 0.001	
	2.0 - < 4.0 years	347 (21.28)	4111 (19.86)	1.74	1.40–2.16	< 0.001	
	4.0 - < 6.0 years	258 (15.82)	3195 (15.43)	1.67	1.33–2.09	< 0.001	
	6.0 - < 8.0 years	225 (13.80)	2573 (12.43)	1.80	1.43–2.27	< 0.001	
	8.0 - < 10.0 years	179 (10.97)	2069 (9.99)	1.78	1.40–2.27	< 0.001	
	10.0 - < 12.0 years	132 (8.09)	1441 (6.96)	1.89	1.46–2.44	< 0.001	
	≥ 12.0 years	109 (6.68)	1641 (7.93)	1.37	1.05–1.79	0.22	
	Unavailable	14 (0.86)	253 (1.22)	1.14	0.65–2.02	0.650	
Sex	Female	689 (42.24)	9851 (47.58)	Base			< 0.001
	Male	939 (57.57)	10,779 (52.07)	1.25	1.12–1.38	< 0.001	
	Unavailable	3 (0.18)	72 (0.35)	0.60	0.19–1.90	0.380	
Neuter	Entire	808 (49.54)	11,353 (54.84)	Base			< 0.001
	Neutered	820 (50.28)	9277 (44.81)	1.24	1.12–1.37	< 0.001	
	Unavailable	3 (0.18)	72 (0.35)	0.59	0.18–1.86	0.365	
Insurance	Non-insured	1329 (81.48)	18,025 (87.07)	Base			< 0.001
	Insured	302 (18.52)	2677 (12.93)	1.53	1.34–1.75	< 0.001	

^a^CI confidence interval

Of the dogs that were not otitis externa cases and with data available on the variable, 15,064 (73.01%) were purebred, 9851 (47.75%) were female and 9277 (44.97%) were neutered. The median adult bodyweight for non-cases was 13.43 kg (IQR: 7.98–24.65, range 1.41–85.00) and the median age was 4.37 years (IQR: 1.85–8.04, range 0.01–20.46). The most common breeds among the non-case dogs were Labrador Retriever (1308, 6.32%), Staffordshire Bull Terrier (1225, 5.92%), Jack Russell Terrier (1148, 5.55%) and Chihuahua (943, 4.56%) along with crossbred dogs (4408, 21.29%) (Tables 1 and 2). Data completeness varied between the variables assessed: breed 99.68%, age 98.80%, sex 99.66%, neuter 99.66% and adult bodyweight 66.82%.

### Predisposing factor analysis

All tested variables except *skull shape* and *Kennel Club recognised breed* were liberally associated with otitis externa in univariable logistic regression modelling and were evaluated using multivariable logistic regression modelling as described in the methods (Tables 1 and 2). The final main breed-focused multivariable model retained five risk factors: *breed, bodyweight relative to breed-sex mean*, *age, sex* and *insurance* (Table 3). No biologically significant interactions were identified. The final model was improved by inclusion of the clinic attended as a random effect (rho: 0.02 indicating that 2% of the variability was accounted for by the clinic attended, *P* = 0.001). The final random effects model showed acceptable model-fit (Hosmer-Lemeshow test statistic: *P* = 0.231) and acceptable discrimination (area under the ROC curve: 0.658).

**Table 3:** Final breed-focused mixed effects multivariable logistic regression model for predisposing factors associated with otitis externa in dogs under primary veterinary care in the VetCompass™ Programme in the UK. Clinic attended was included as a random effect

Variable	Category	Odds ratio	95% CI^a^	*P*-value
Breed	Crossbreed	Base		
	Basset Hound	5.87	3.26–10.57	< 0.001
	Chinese Shar Pei	3.44	2.04–5.78	< 0.001
	Labradoodle	2.95	1.96–4.46	< 0.001
	Beagle	2.54	1.67–3.86	< 0.001
	Golden Retriever	2.23	1.51–3.28	< 0.001
	Cockapoo	2.22	1.65–3.00	< 0.001
	American Bulldog	2.16	1.09–4.26	0.027
	French Bulldog	2.11	1.50–2.96	< 0.001
	English Bulldog	2.08	1.35–3.21	0.001
	Pug	1.95	1.40–2.72	< 0.001
	Cavapoo	1.92	1.03–3.59	0.040
	West Highland White Terrier	1.72	1.27–2.34	< 0.001
	Rottweiler	1.67	1.01–2.77	0.047
	Cocker Spaniel	1.67	1.29–2.17	< 0.001
	Labrador Retriever	1.64	1.33–2.01	< 0.001
	Bichon Frise	1.49	1.02–2.18	0.042
	Boxer	1.40	0.89–2.22	0.149
	English Springer Spaniel	1.24	0.88–1.76	0.226
	Lhasa Apso	1.23	0.80–1.89	0.351
	Border Terrier	1.15	0.71–1.85	0.571
	German Shepherd Dog	1.13	0.80–1.61	0.482
	Cavalier King Charles Spaniel	1.13	0.78–1.64	0.518
	Shih Tzu	1.00	0.73–1.36	0.986
	Other purebreds	0.96	0.80–1.14	0.625
	Staffordshire Bull Terrier	0.95	0.73–1.23	0.678
	Jack Russell Terrier	0.52	0.37–0.72	< 0.001
	Yorkshire Terrier	0.49	0.32–0.74	0.001
	Border Collie	0.34	0.19–0.58	< 0.001
	Chihuahua	0.20	0.11–0.36	< 0.001
Bodyweight relative to breed mean	Lower	Base		
	Equal/Higher	1.45	1.29–1.63	< 0.001
	Unavailable	0.80	0.69–0.93	0.003
Age (years)	< 1.0 years	1.00		
	1.0 - < 2.0 years	1.56	1.24–1.97	< 0.001
	2.0 - < 4.0 years	1.43	1.13–1.80	0.003
	4.0 - < 6.0 years	1.38	1.08–1.76	0.010
	6.0 - < 8.0 years	1.47	1.14–1.89	0.003
	8.0 - < 10.0 years	1.45	1.11–1.88	0.006
	10.0 - < 12.0 years	1.57	1.19–2.07	0.001
	> or = 12.0 years	1.22	0.92–1.63	0.171
	No age available	1.44	0.80–2.58	0.220
Sex	Female	Base		
	Male	1.21	1.09–1.34	< 0.001
	Unrecorded	0.78	0.24–2.54	0.683
Insurance	Uninsured	Base		
	Insured	1.34	1.17–1.54	< 0.001

^a^CI Confidence interval

After accounting for the effects of the other variables evaluated, 16 breeds showed increased odds of otitis externa compared with crossbred dogs. The breed types with the highest odds included Basset Hound (odds ratio [OR] 5.87, 95% CI 3.26–10.57, *P* <  0.001), Chinese Shar Pei (OR 3.44, 95% CI 2.04–5.78, *P* <  0.001), Labradoodle (OR 2.95, 95% CI 1.96–4.46, *P* <  0.001), Beagle (OR 2.54, 95% CI 1.67–3.86, *P* <  0.001) and Golden Retriever (OR 2.23, 95% CI 1.51–3.28, *P* <  0.001). Four breeds showed reduced odds of otitis externa compared with crossbreds: Chihuahua (OR: 0.20, 95% CI 0.11–0.36, *P* <  0.001), Border Collie (OR: 0.34, 95% CI 0.19–0.58, *P* <  0.001), Yorkshire Terrier (OR: 0.49, 95% CI 0.32–0.74, *P* = 0.001) and Jack Russell Terrier (OR: 0.52, 95% CI 0.37–0.72, *P* = 0.001). Individual dogs with an adult bodyweight that was equal or higher than their breed/sex mean had 1.45 (95% CI 1.29–1.63, *P* <  0.001) times the odds of otitis externa compared with dogs that weighed below their breed/sex mean. All age groups over 1 year showed higher odds of otitis externa compared with dogs aged under 1 year. Males had 1.21 times the odds (95% CI 1.09–1.34, *P* <  0.001) of otitis externa compared with females. Insured dogs had 1.34 (95% CI 1.17–1.54, *P* <  0.001) times the odds of otitis externa compared with uninsured dogs (Table 3).

As described in the methods, variables derived from the breed information individually replaced *breed* in the final breed-focused model. Designer types had 1.63 times the odds (95% CI 1.31–2.03, *P* <  0.001) of otitis externa compared with crossbred dogs. Gundog (1.42 OR, 95% CI 1.23–1.65, *P* <  0.001) and Utility (1.25 OR, 95% CI 1.05–1.47, *P* = 0.011) Kennel Club breed groups showed higher odds of otitis externa compared with breeds that are not recognized by the Kennel Club, while Pastoral (0.67 OR, 95% CI 0.52–0.88, *P* = 0.004) and Toy (0.68 OR, 95% CI 0.56–0.81, *P* <  0.001) showed lower odds. Compared with breeds with erect ear carriage, breeds with pendulous ear carriage had 1.76 times the odds (95% CI 1.48–2.10, *P* <  0.001) and dogs with V-shaped drop ear carriage had 1.84 times the odds (95% CI 1.53–2.21, *P* <  0.001) of otitis externa. Poodle types had 1.91 times the odds (95% CI 1.57–2.32, *P* <  0.001) of otitis externa compared with non-poodle types. Spaniel types had 1.24 times the odds (95% CI 1.05–1.47, *P* = 0.013) of otitis externa compared with non-spaniel types. Dogs weighing under 10 kg had lower odds of otitis externa than all other categories with higher bodyweight. Skull shape was not associated with otitis externa (Table 4).

**Table 4:** Results for risk factors that directly replaced the breed variable in the final breed-focused mixed effects multivariable logistic regression model (along with age, bodyweight relative to breed mean, sex and insurance status). Adult (> 18 months) bodyweight (kg) replaced the breed and bodyweight relative to breed mean variables in the final breed-focused mixed effects multivariable logistic regression model. These results report associations between these predisposing factors and otitis externa in dogs under primary veterinary care in the VetCompass™ Programme in the UK. Clinic attended was included as a random effect

Variable	Category	Odds ratio	95% CI^a^	Category *P*-value
Purebred status	Crossbred	Base		
	Designer	1.63	1.31–2.03	< 0.001
	Purebred	1.13	0.99–1.29	0.060
Kennel Club Breed Group	Breed not Kennel Club recognised	Base		
	Toy	0.68	0.56–0.81	< 0.001
	Utility	1.25	1.05–1.47	0.011
	Terrier	0.88	0.75–1.04	0.143
	Gundog	1.42	1.23–1.65	< 0.001
	Hound	1.23	0.94–1.60	0.128
	Pastoral	0.67	0.52–0.88	0.004
	Working	0.94	0.72–1.24	0.672
Ear carriage	Erect	Base		
	Semi-erect	1.03	0.86–1.24	0.757
	V-shaped drop	1.84	1.53–2.21	< 0.001
	Pendulous	1.76	1.48–2.10	< 0.001
	Variation	1.36	1.15–1.60	< 0.001
Poodle	Not poodle-type	Base		
	Poodle-type	1.91	1.57–2.32	< 0.001
	Not categorised	0.90	0.79–1.03	0.117
Spaniel	Non spaniel-type	Base		
	Spaniel-type	1.24	1.05–1.47	0.013
	Not categorised	0.88	0.77–1.00	0.054
Adult (> 18 months) bodyweight (kg)	< 10.0	Base		
	10.0 - < 15.0	1.97	1.66–2.35	< 0.001
	15.0 - < 20.0	1.58	1.28–1.94	< 0.001
	20.0 - < 25.0	1.65	1.34–2.04	< 0.001
	25.0 - < 30.0	1.59	1.27–1.99	< 0.001
	30.0 - < 40.0	2.05	1.69–2.48	< 0.001
	≥ 40.0	2.35	1.80–3.07	< 0.001
	Unavailable	0.99	0.83–1.17	0.874

^a^CI Confidence interval

## Discussion

This is the largest study of dogs under primary veterinary care to date that provides epidemiological evidence on the frequency of diagnosis of otitis externa and its predisposing factors. Following some prior published evidence [8, 26, 35], the current study explored the odds of otitis externa between canine breeds with differing forms of ear carriage as a predisposing factor. In this rather large current cohort of cases, our results show that breeds with pendulous pinnal carriage had 1.76 times the odds of otitis externa compared with breeds with erect carriage. However, the results also provided some novel insights to show that dogs with V-shaped drop pinna have similar risk of otitis externa as breeds with pendulous ears (OR 1.84). Conversely, no difference in odds was detected between dogs with erect compared to dogs with semi-erect pinnae. Pendulous pinnal carriage has long been reported as a predisposing factor for otitis externa [8, 26, 35] with the suggestion that this conformation can result in heat and moisture retention within the ear canal [26] and may be more likely to retain foreign material than other ear carriage types (Griffin 2010, Miller 2013). However, the identification of V-shaped drop pinna as a predisposing factor for otitis externa with a similar effect to pendulous ears is a novel finding. Future studies to explore possible differences between the pathogenetic pathways for otitis externa in pendulous and V-shaped pinnal conformations are warranted, particularly to link this novel predisposing factor information with primary (e.g., allergic, endocrine, foreign bodies), secondary (variability of infectious agents) and perpetuating (such as otitis media, chronic tympanic membrane and external canal changes) factors [8]. It should be noted that ear carriage and breed are closely linked concepts and therefore it is challenging to unravel the relative contributions of predisposing factors (such as ear carriage) from primary factors (such as allergic dermatitis) in breeds with a propensity to both. Hence the current study took a quantitative study design approach with multivariable modelling to report overall effects of predisposing factors at a population level but acknowledges that there will be additional nuance at the individual animal level that breeders, welfare scientists and veterinarians will need to additionally consider. However, notwithstanding these considerations about crossover of effects between differing PSPP factors, the current study provides strong epidemiological evidence that pinnal carriage per se acts as a predisposing factor for otitis externa. This information could support the Kennel Club Breed Health and Conservation Plans, which aims to identify, prioritise and advise on breed health concerns [11].

After accounting for confounding effects, Basset Hound (OR 5.87), Chinese Shar Pei (OR 3.44), Labradoodle (OR 2.95), Beagle (OR 2.54) and Golden Retriever (OR 2.23) had the highest odds of otitis externa compared with crossbreeds. Basset Hounds have previously been reported at higher risk of otitis externa due to their highly pendulous pinnae, a phenotype that was selected supposedly to lead scents to their noses [36, 37]. Although without a firm evidence base, it is also possible that the long and deep ear canal in Basset Hounds may also act to delay resolution of infections once started, and lead to higher probability of otic chronicity in this breed. The current study reported prevalence (i.e. the proportion of dogs diagnosed at least once during the study year) but did not take direct account of the severity or duration of these clinical events [29]. Basset Hounds are reported to harbour more *Malassezia* spp. yeasts on their skin, including within the ear canal, compared with toy breeds [38]. This may manifest clinically as dark staining of the skin with or without discharge in their ear canals and skin folds and can be verified by cytology [39, 40]. However, without accompanying erythema and/or pruritus, this presentation should not necessarily lead to a diagnosis of otitis externa [38]. This highlights the importance of thorough examination of the ear canal in combination with the pinna (and the overall body skin) as well as assessing for clinical signs (e.g., pruritus, head shaking) on the path of the decision-making process towards a diagnosis of otitis externa. Beagles and Golden Retrievers have dropped (pendulous or V-shaped) pinnae which, as identified in the current study, may increase the risk of these breeds for otitis externa [41]. Chinese Shar Pei, however, have semi-erect ears, suggesting an alternative pathogenesis in this breed. Following selective breeding to achieve the Chinese Shar Pei breed standard, hyaluronic acid accumulates in their dermis resulting in folded and narrowed ear canals, predominantly of the vertical part [42–44]. Breed health plans for breeds at high risk of otitis externa could consider prioritising control of otitis externa as a priority health goal [11] while owners and veterinarians should be especially vigilant about ear examination and care in such predisposed breeds.

For the first time, we report here on protection (i.e. reduced odds) for otitis externa in the Chihuahua (OR 0.20), Border Collie (OR 0.34), Yorkshire Terrier (0.49) and Jack Russell Terrier (0.52). To date, the veterinary literature has focused mainly on breed predisposition to disease [45]. However, there is an increasing awareness of the value of exploring protection to disorders within breeds with a view to elucidating novel genetic, conformational and aetiopathogenetic pathways for reduced disorder occurrence [10, 21]. Discovery of protected breeds could also support plans to improve breed health and reduce disorder incidence in at-risk breeds by outcrossing programmes [46, 47]. Chihuahua was the breed with the lowest risk to otitis externa, potentially due, in part, to their erect pinnal conformation [8, 41], low body weight (smallest dog breed) and low tendency to primary skin disease in general [48]. The presence of guard hairs at the entrance to the ear canal in Chihuahuas may also contribute to a low risk of otitis externa. However, there is controversy about whether guard hairs act more to protect ear canals from the ingress of foreign material or to prevent the egress of foreign material from the ear canal. Preventing egress would likely delay resolution of otitis externa. For example, although German Shepherd Dog and Siberian Husky breeds both have plentiful guard hairs at the entrance of the ear canal, the former appears to be more often affected by otitis externa than the latter, suggesting that the role of guard hairs in otitis externa may be quite complex [49]. Other than Border Collies, it is notable that the breeds identified as significantly protected were all small breed dogs, suggesting that small body size and therefore shorter ear canals [4] in addition to pinnal conformation may be contributory protective factors [41].

The current study is one of the first to explore disorder occurrence across a range of designer breed-types. A hybrid vigour effect has been previously proposed whereby designer dogs with greater outbreeding are expected to show better general health than pedigree dogs that are more inbred [50]. However, this effect is not supported by the current study for a polygenic disorder such as otitis externa where some common designers types even showed predispositions to increased disorder risk. Designer breeds overall showed 1.63 times the odds of otitis externa compared with crossbreeds. The higher odds identified may be less to do with being designer per se and more to do with the poodle or spaniel component that is common among popular designer types. In line with this, Labradoodles (OR 2.95), Cockapoos (OR 2.22) and Cavapoos (OR 1.92) were all identified at greater risk of otitis externa compared with crossbreeds. Poodle-types (OR 1.91) showed greater risk of otitis externa compared with non-poodle types while spaniel-types (OR 1.24) had greater risk than non-spaniel types. Increased risk for otitis externa in poodles may in part be due to their pendulous pinnal carriage, excessive curly hairs in the external ear canal [1, 26, 51], aural microclimate and the proposed need for repeated ear plucking that is under heavy controversy [25]. In addition, poodles are commonly affected by allergic skin disease that can act as a primary cause of otitis externa [52] and especially prone to *Malassezia* overgrowth in the ear canals [22]. The poodle breed itself is a water hound which enhances the behavior trait for excessive swimming, thus a higher likelihood of moist ear canals and Swimmer’s ear [25]. This poodle predisposition may even be increased by crossing a poodle with a spaniel which is another predisposed breed type with pendulous pinnal carriage, or with a breed such as the Labrador Retriever that is prone to primary skin disease contributing to aural atopic disease [53–55]. Therefore, breeders of designer-types need to be wary to avoid selecting towards a phenotype that combines differing risk factors from parental breeds and therefore could increase disease risk in the first generation of puppies (often called the F1 hybrids) [56, 57]. The wider variability in the phenotypes of progeny from planned hybridisation between different breeds may also lead to greater variability in the health status across individual dogs from these hybridisation breeding programmes.

It is worth noting that the current study was based on dogs that are under veterinary care in the UK [34]. In consequence, these dogs reflect the wider dog population of the UK but there may be differences in the typical conformations between the estimated subsets of around 30% within breeds that are registered with The Kennel Club and the remaining estimated 70% that are not registered with The Kennel Club [58]. Similarly, there may be variation in typical breed conformations, genetics and canine lifestyle factors between countries and therefore extrapolation of the results in the current paper should be taken cautiously [59–61].

Bodyweight was identified as a predisposing factor for otitis externa, with dogs weighing over 40 kg at greatest risk of otitis externa (OR 2.35) compared with dogs under 10 kg. Breed and bodyweight are highly correlated, therefore these two factors were not included in the same models [62]. However, this increased risk with increasing absolute bodyweight is also reflected in the current results by an increased risk in higher weight-carrying dogs within the same breed. Dogs weighing at or above the breed mean bodyweight had 1.45 times the odds of otitis externa compared with those weighing below. It is possible that increased risk of otitis externa in heavier dogs may in part be related to obesity and neutering state, but further studies are needed to elucidate this mechanism more fully. Nonetheless, these suggestive findings that obesity may be linked to higher odds of otitis externa should further promote the importance of maintaining a moderate body condition score in dogs [63].

Sex was identified as a weak but still predisposing factor for otitis externa in the current study, with males at 1.21 times the odds of otitis externa compared with females. A report based on 273 dogs presenting to teaching and referral hospitals in India similarly identified higher prevalence of otitis externa in male dogs compared to females [24]. Several UK breed-based reports failed to identify sex-related differences for otitis externa [15, 17, 55, 64]. However, a significantly higher prevalence of otitis externa in males compared to females has been reported in the West Highland White Terrier [16] and Chihuahua [65]. Androgen hormones may increase sebum production, which is a predisposing factor to flare up of latent otic infections as well as favouring *Malassezia* spp. overgrowth. Conversely, oestrogens elicit an opposite response of drying the skin that may promote secondary infections, especially in allergic dermatitis cases [7, 24, 25]. The current study provides some evidence of an increased risk in males overall but further research is required to determine if this is universal across breeds or whether this effect varies between breeds and is moderated by other factors.

No substantial associations were identified between variants of skull conformation and otitis externa in the current study. Some increased risk may have been expected in brachycephalic breeds from a biological perspective because the external ear canal of brachycephalic dogs is often folded and narrowed due to the skull shape, which would be expected to promote reduced air flow and increased humidity in the ear canal, thus promoting and prolonging otitis externa [66]. Consistent with this rationale, the current study showed that breeds with more extreme brachycephaly, such as French Bulldogs, English Bulldogs and Pugs, had greater odds of otitis externa than crossbreeds. There is conformational variation both within, and between, brachycephalic breeds [67] and therefore it may be that skull shape acts as a predisposing factor only for the more extreme brachycephalic breed types. This effect may act as another reason to support the increasing calls to reduce the degree of extreme exaggeration in many brachycephalic breeds [68, 69].

This study focused on predisposing factors that centre on breed and other demographic characteristics. This epidemiological approach is in line with the critical data gaps on population-based breed prevalence predispositions that have been identified especially over the past 15 years in relation to improving the genetic health of purebred dogs [70–72]. The current study aims to build on an expanding literature on breed-related studies that are filling this data gap [21, 73–76] and that are supporting reforms to breed health such as The Kennel Club’s Breed Health and Conservation Plans project [11]. However, there are other categories of risk factors that could have also been considered and that would have added additional useful inference from other perspectives. There is little information available at a population level about the relative proportional contributions of other underlying conditions (e.g., atopic dermatitis, aural foreign body) to the overall disorder burden from otitis externa. Exploration of the comorbid presence of a range of potential clinical risk factors in each of the otitis externa cases in the current dataset could provide some information on the relative importance of each of these to the overall occurrence of otitis externa in dogs and therefore constitutes a useful concept for future study [74]. Extraction of detailed data on veterinary clinical management and therapy offers another research approach that could build on the current data to contribute to a deeper understanding of the pathophysiology of these otitis externa cases at a population level [77]. Elucidation of proportional usage of antibiosis, for example, could provide evidence of a bacterial role in the otitis externa process while evidence from bacterial culture and antimicrobial sensitivity testing could provide deeper insights into the most commonly associated bacteria and their common antibiograms [78, 79]. There is also currently high interest in exploring aspects related to antimicrobial stewardship in companion animals and therefore benchmarking of current first opinion therapeutic patterns to offer additional scientific benefits [75, 80]. A deeper understanding of breed as a risk factor for otitis externa could take into account the severity of the disorder phenotype experienced by these dogs as well as a range of temporal characteristics including the duration of individual events, recurrence and chronicity. Analysis of these features has been applied previously to compare welfare impact across common disorders in dogs but this approach could also be taken to compare between breeds in future studies [29].

Research based on primary-care data offers novel opportunities to better understand common and less complicated disorders [14] but the methodology does have some important limitations which have been previously documented [14, 81]. In addition to these, the current study may have under-estimated the true prevalence of otitis externa because some owners of affected dogs may not have sought veterinary attention e.g., for financial reasons: this possibility is suggested by higher odds of otitis externa in insured dogs in the current study. Additionally, some owners may not have acknowledged the existence of otitis externa in their dog because of limited awareness of the clinical signs or the welfare significance of aural pain and hearing alterations [28–31]. Dissemination of information from veterinarians on the need for increased aural vigilance by owners of breeds with known predisposing factors for otitis externa may therefore assist with earlier recognition and higher levels of presentation for veterinary care of affected dogs. Additional breeds to the ones included in the current study have previously been reported with predisposition to otitis externa [8, 82] but there were insufficient numbers for many of these rarer breeds in the current study for reliable assessment.

## Conclusions

This large study using primary-care veterinary data reports a 7.30% prevalence for otitis externa in dogs in the UK, highlighting the importance of otitis externa to canine welfare and clinical caseloads. Conformational predispositions were identified, with dogs with pendulous and V-shaped drop pinnal carriage at higher risk of otitis externa than dogs with erect pinnal carriage. Strong breed effects as predisposing factors were identified, with Basset Hound, Chinese Shar Pei, Labradoodle, Beagle and Golden Retriever showing greatest predisposition to otitis externa. In addition, designer breed types had higher odds of otitis externa compared with crossbreeds. Awareness of these risk factors could assist veterinary practitioners and owners to reduce the contribution of aural disorders to the overall welfare burden in dogs by improved selection of dogs for breeding and ownership, and by earlier recognition of clinical events of otitis externa. Greater understanding on how breed itself and breed-associated ear carriage conformation factors affect the probability of otitis externa in dogs can help veterinary practitioners to promote cautious and low-harm approaches to preventing otitis externa (e.g., by advocating ear cleaning with a dry paper cloth, judicious use of ear cleaners with antimicrobial properties and avoidance of overzealous ear-cleaning) and can also assist breeders to breed away from features of dogs that predispose to otitis externa. Owners can be encouraged to regularly check their dog’s ears for malodour and exudate, and veterinarians can follow up by otoscopy and cytological examination. Breed health plans could consider inclusion of otitis externa as a priority condition in predisposed breeds and conformations.

## Methods

The study population included all dogs under primary veterinary care at clinics participating in the VetCompass Programme during 2016. Dogs under veterinary care were defined as having either a) at least one electronic patient record (EPR) (free-text clinical note, treatment or bodyweight) recorded during 2016 or b) at least one EPR recorded during both 2015 and 2017. VetCompass collates de-identified EPR data from primary-care veterinary practices in the UK for epidemiological research [34]. Data fields available to VetCompass researchers include a unique animal identifier along with species, breed, date of birth, sex, neuter status and insurance. Clinical information from free-form text clinical notes, summary diagnosis terms [83], bodyweights and treatment with relevant dates were also available.

A cohort study design was used to estimate the one-year (2016) period prevalence of otitis externa and to explore associations with signalment and conformation as predisposing factors. Sample size calculations estimated that 13,621 dogs would need to be assessed to estimate prevalence for a disorder occurring in 10.0% of dogs [84] with 0.5% acceptable margin of error at a 95% confidence level from a population of 905,544 dogs [85]. Ethics approval was obtained from the RVC Ethics and Welfare Committee (reference SR2018–1652).

The case definition for otitis externa cases required evidence in the clinical records that otitis externa was diagnosed to exist as a clinical condition at some point during 2016. The clinical decision-making process was completely at the discretion of the attending veterinary surgeons. The clinical records of a randomly selected subset of dogs from the sampling frame of unique dogs in the overall study population were reviewed manually in detail to identify all dogs that met the case definition for otitis externa [84]. This study aimed to identify and extract information on the diagnosed cases rather than to question how these diagnoses were made. No additional information was extracted on laterality, chronicity or comorbidity with other conditions. No distinction was made between pre-existing and incident cases of otitis externa.

Breed descriptive information entered by the participating practices was cleaned and mapped to a VetCompass breed list derived and extended from the VeNom Coding breed list that included both recognised purebred breeds and also designer breed terms [83]. A *purebred* variable categorised all dogs of recognisable breeds as ‘purebred’, dogs with contrived breed names generated from two or more purebred breed terms as designers (e.g., Labradoodle) and all remaining dogs with breed information as ‘crossbred’ [59]. A *breed* variable included individual pure breeds and designers represented by over 300 dogs in the overall study population or with ≥10 otitis externa cases, a grouped category of all remaining purebreds and a grouping of general crossbred dogs. This approach was taken to facilitate statistical power for the individual breed analyses [86]. Crossbreeds were used as the comparator group because they were the largest single group.

Breeds were characterised by ear carriage based on pinnal phenotypes typically described for each breed [60, 87, 88]. The categories of ear carriage included erect (also known as prick or upright e.g., German Shepherd Dog), semi-erect (also known as cocked or semi-pricked e.g., Rough Collie), V-shaped drop (also known as folded e.g., Hungarian Vizsla), pendulous (also known as drop or pendant, e.g., Basset Hound) and unspecified. Based on various kennel club breed descriptions [59, 89], breeds were also characterised by skull shape (dolichocephalic, mesocephalic, brachycephalic, not categorised), spaniel (spaniel, non-spaniel, not categorised) and poodle (poodle, non-poodle, not categorised) status for analysis. Crossbreds were classified as ‘not categorised’ for these variables. A *Kennel Club breed group* variable classified breeds recognised by the UK Kennel Club into their relevant breed groups (Gundog, Hound, Pastoral, Terrier, Toy, Utility and Working) and all remaining types were classified as non-Kennel Club recognised [59].

Neuter and insurance status were defined by the final available EPR value. Adult bodyweight was defined as the mean of all bodyweight (kg) values recorded for each dog after reaching 18 months old and was categorised as: < 10.0, 10.0 to < 15.0, 15.0 to < 20.0, 20.0 to < 25.0, 25.0 to < 30.0, 30.0 to < 40.0 and ≥ 40.0. Mean adult bodyweight was generated for all breed/sex combinations with adult bodyweight available for at least 100 dogs in the overall study population and used to categorise individual dogs as “at or above the breed/sex mean”, “below the breed/sex mean” and “no recorded bodyweight”. Age (years) was defined at December 31, 2016 and was categorised as: ≤ 1.0, 1.0 to < 2.0, 2.0 to < 4.0, 4.0 to < 6.0, 6.0 to < 8.0, 8.0 to < 10.0, 10.0 to < 12.0 and ≥ 12.0.

Following internal validity checking and data cleaning in Excel (Microsoft Office Excel 2013, Microsoft Corp.), analyses were conducted using Stata Version 13 (Stata Corporation).

One-year period prevalence values with 95% confidence intervals (CI) described the probability of diagnosis at least once during 2016 in dogs overall and in common breeds. The CI estimates were derived from standard errors based on approximation to the binomial distribution [90]. Predisposing factor analysis included dogs with otitis externa as cases and all remaining dogs as non-cases. Binary logistic regression modelling was used to evaluate univariable associations between risk factors of interest as potential predisposing factors and also factors that were included to account for confounding (*breed, ear carriage, skull shape, spaniel, poodle, purebred, Kennel Club recognised breed, Kennel Club breed group, adult bodyweight, bodyweight relative to breed/sex mean, age, sex, neuter* and *insurance*) with an outcome variable of otitis externa during 2016. Because breed was a factor of primary interest as a predisposing factor, variables derived from breed information were considered as correlated with breed (*ear carriage, skull shape, spaniel, poodle, purebred, Kennel Club recognised breed* and *Kennel Club breed group*) and were excluded from initial breed multivariable modelling. Instead, each of these variables individually replaced the *breed* variable in the main final breed-focused model to evaluate their effects after taking account of the other variables. *Adult bodyweight* (a defining characteristic of individual breeds) replaced *breed* and *bodyweight relative to breed/sex mean* in the final breed-focused model. Risk factors with liberal associations in univariable modelling (*P* <  0.2) were taken forward for multivariable evaluation. Model development used manual backwards stepwise elimination. Clinic attended was evaluated as a random effect and pair-wise interaction effects were evaluated for the final model variables [20]. The area under the ROC curve and the Hosmer-Lemeshow test were used to evaluate the quality of the model fit and discrimination (non-random effect model) [20, 91]. Statistical significance was set at *P* <  0.05.

## Data Availability

The datasets generated during and/or analysed during the current study are available at the RVC Research Online repository https://researchonline.rvc.ac.uk/id/eprint/13333

## Abbreviations

CI: Confidence interval
EPR: Electronic patient record
IQR: Interquartile range
KC: The Kennel Club
OR: Odds ratio
